# Synthesis of an Azido-Substituted 8-Membered Ring Laddersiloxane and Its Application in Catalysis

**DOI:** 10.3390/molecules30020373

**Published:** 2025-01-17

**Authors:** Yujia Liu, Niyaz Yagafarov, Koki Shimamura, Nobuhiro Takeda, Masafumi Unno, Armelle Ouali

**Affiliations:** 1 Department of Chemistry and Chemical Biology, Graduate School of Science and Technology, Gunma University, 1-5-1 Tenjin-cho, Kiryu 376-8515, Japan; driagafarov@gunma-u.ac.jp (N.Y.); t241a052@gunma-u.ac.jp (K.S.); ntakeda@gunma-u.ac.jp (N.T.); 2 ICGM, Univ Montpellier, CNRS, ENSCM (Institut Charles Gerhardt Montpellier, Université de Montpellier, Centre National de la Recherche Scientifique, École Nationale Supérieure de Chimie de Montpellier), 1919 Route de Mende, CEDEX 05, 34293 Montpellier, France

**Keywords:** tricyclic laddersiloxanes, azido-substituted laddersiloxane, click chemistry, catalysis, oxidative dehydrogenation of alcohols

## Abstract

A first *syn*-type tricyclic 8-8-8 (three fused-8-membered ring) laddersiloxane functionalized with four azido groups was successfully synthesized through efficient and highly selective hydrosilylation and nucleophilic substitution, achieving an excellent overall yield. The starting material, a tetravinyl-substituted 8-8-8 laddersiloxane, was prepared via a straightforward and scalable method. The obtained azido-functionalized ladder compound, fully characterized, constitutes a versatile building block for hybrid materials. Reacting this compound with 2-ethynylpyridine via click chemistry yielded a multidentate ligand containing four 2-triazole-pyridyl moieties. This N,N-bidentate ligand was subsequently employed in copper-catalyzed alcohol oxidative dehydrogenation reactions, demonstrating its potential in catalysis.

## 1. Introduction

Silsesquioxanes (SQs) are a class of siloxane compounds predominantly composed of silicon atoms bonded to three oxygen atoms and one organic group. These compounds exhibit remarkable thermal stability and mechanical strength due to the abundant silicon–oxygen bonds. Additionally, the organic substituents attached to the silicon atoms facilitate the introduction of various functional groups, enabling fine-tuning of their properties. This organic–inorganic hybrid property makes SQ compounds attractive precursors for the synthesis of novel hybrid materials [1,2,3,4,5,6,7,8].

Recent research into SQ compounds has led to significant advancements, particularly in the development of well-defined cage- [3,4,5,6,7,8], double-decker- [9], and ladder-type structures [10]. These structures offer enhanced thermal, mechanical and electronic properties compared to random forms. Among these, ladder-type silsesquioxanes (also known as laddersiloxanes), characterized by highly ordered double-chain architectures and the highest refractive index among well-defined SQ compounds, present potential in optical and electronic applications [10,11]. Following Brown’s initial proposition of the ladder structure [12], regulated synthetic methods for producing well-structured laddersiloxanes have been developed [10].

The most studied laddersiloxanes are *syn*-type tricyclic 6-8-6 and 8-8-8 compounds, which feature fused 6-8-6- and 8-8-8-membered rings. A common synthetic route involves the reaction of all-*cis*-cyclotetrasiloxanetetraol derivatives with dichlorosilanes or dichlorosiloxanes in the presence of a base (e.g., pyridine or triethylamine), yielding *syn*-type tricyclic laddersiloxanes with various functional groups, including alkyl, aryl, and alkenyl groups [10,13,14,15,16,17]. Furthermore, the functionalization of alkenyl groups allows the introduction of chloro, iodo, and thiol groups into the tricyclic ladder framework while retaining the *syn*-type conformation [14,16,17,18]. Additionally, *syn*-type 8-8-8 laddersiloxanes can be synthesized from all-*cis* cyclic siloxane via intramolecular condensation in the presence of hydrochloric acid and tris(pentafluorophenyl)borane (B(C_6_F_5_)_3_) [19,20], or from *cis,trans,cis*-cyclic siloxane and tetraphenyldisilanol [21], or by oxidation of the stereo-regulated tricyclic laddersilanes [22]. The functionalized *syn*-type tricyclic laddersiloxanes have potential applications as precursors for hybrid nanoporous materials in wastewater treatment [23], for the synthesis of conjugated oligo-/polymers in semiconducting materials [24], and for the preparation of dendrimers and Janus-type materials [17].

Among the various possible functional groups, azido groups are highly reactive and can undergo copper-catalyzed Huisgen [3+2] cycloaddition (CuAAC) with alkynes. This strategy related to click chemistry [25] represents a powerful method for modifying well-defined SQ compounds to create a variety of functional materials, such as recyclable catalysts [26,27,28,29], bioactive compounds [30,31,32,33,34], optoelectronic devices [35,36]. However, tricyclic laddersiloxanes with azido groups have not been reported previously, and we believe they hold great promise as precursors for a diverse range of applications.

In this work, we report a high-yielding synthesis of the first *syn*-type tricyclic 8-8-8 laddersiloxane with four azido groups, starting from a newly developed tetrachloro-functionalized compound. The tetraazido laddersiloxane was then used to synthesize an unprecedented laddersiloxane featuring four triazole-pyridyl moieties via click chemistry. This compound was subsequently employed as a ligand in the copper-catalyzed oxidative dehydrogenation of alcohols.

## 2. Results and Discussion

### 2.1. Synthesis of 8-8-8 Laddersiloxane with Four Azido Groups

**Scheme 1:**
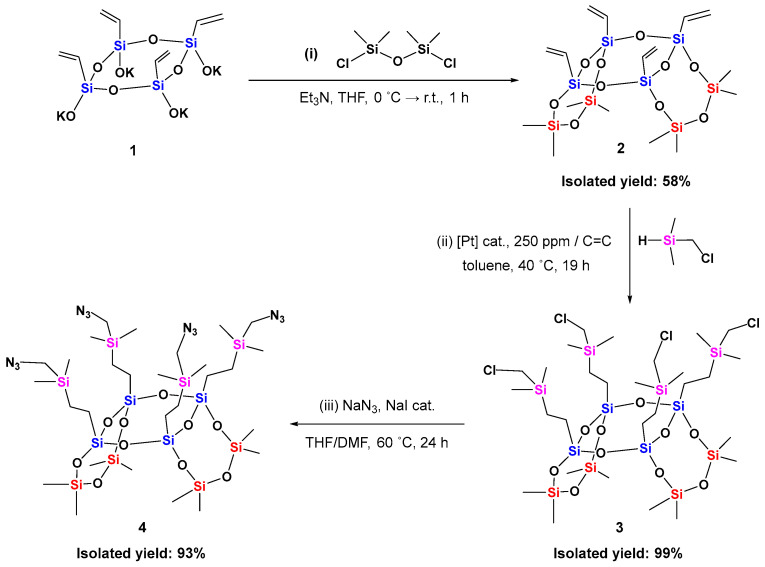
Synthetic route of *syn*-type tricyclic 8-8-8 laddersiloxane (**4**) with four azido groups.

The *syn*-type tricyclic 8-8-8 laddersiloxane with four vinyl groups (compound **2**) was previously synthesized in a total yield of 50% from all-*cis*-tetravinylcyclotetrasiloxanolate (**1**) via a two-step process: silylation with chlorodimethylsilane, followed by intramolecular cyclization in the presence of water, using B(C_6_F_5_)_3_ as a catalyst [20]. In this study, we developed a simplified one-step synthesis, starting from potassium all-*cis*-tetravinylcyclotetrasiloxanolate (**1**) and using commercially available 1,3-dichloro-1,1,3,3-tetramethyldisiloxane (Scheme 1, **i**). Precursor **1** was freshly prepared through controlled hydrolysis of tetraethoxyvinylsilane following a reported procedure [37], and thoroughly dried prior to use.

For the synthesis of 8-8-8 laddersiloxane, well-dried compound **1**, distilled triethylamine, and anhydrous THF were introduced in an argon-purged Schlenk flask, then the mixture was cooled to 0 °C. A solution of 1,3-dichloro-1,1,3,3-tetramethyldisiloxane in dry THF was slowly added dropwise at 0 °C under an argon atmosphere. After addition, the mixture was stirred for an additional hour at room temperature. The desired tetravinyl laddersiloxane (**2**) was obtained in good yield through simple extraction and evaporation, requiring no further purification (Scheme 1, **i**). Notably, this synthetic route is a single-step procedure that eliminates the need for an expensive and water-sensitive borane catalyst, achieving a higher yield (58%) that is comparable to the previously reported analogous laddersiloxanes [13,14,15,16,17]. The *syn*-type structure of compound **2** was confirmed by ^1^H, ^29^Si NMR and mass spectroscopies, with results consistent with the literature data [20].

**Figure 1:**
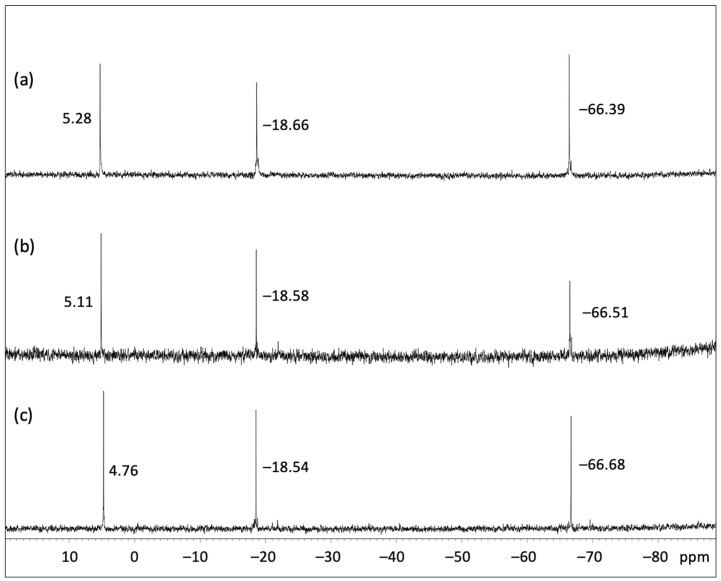
^29^Si NMR spectra (CDCl_3_) of laddersiloxanes **3** (**a**), **4** (**b**), and **5** (**c**).

Next, the hydrosilylation of tetravinyl laddersiloxane **2** with (chloromethyl)dimethylsilane was carried out at 40 °C (Scheme 1, **ii**) using a very small amount of Karstedt’s catalyst (250 ppm of Pt per C=C bond). This reaction quantitatively and selectively yielded *β*-product **3**, which was isolated in a quantitative yield using a simple silica plug without further purification. This result aligns with the previously reported hydrosilylation of *syn*-type tricyclic laddersiloxanes [14,15,16,17]. The ^29^Si NMR spectrum of tetrachloro laddersiloxane **3** displayed three singlets: a resonance at –66.39 ppm assigned to the bridged silicon atoms (T-unit Si in blue), a resonance at –18.66 ppm corresponding to the side silicon atoms (D-unit Si in red), and a resonance at 5.28 ppm contributing to the carbosilane units (Si in pink) (Figure 1a). The chemical shifts of the T-unit Si and carbosilane units in compound **3** are consistent with those previously reported for analogous chloro-functionalized 6-8-6 laddersiloxanes [16,17], while the chemical shifts of the D-unit Si fall within the same range as those reported for 8-8-8 laddersiloxanes [20]. The structure of compound **3** was further confirmed by ^1^H, ^13^C NMR, infrared spectroscopy and elemental analysis (see Supplementary Materials, Figures S1, S2 and S10). MALDI-TOF mass spectrometry showed an experimental mass of 1068.88 g/mol for **3** [M+Na]^+^, which matches well with the calculated mass of 1069.10 g/mol (Figure S13). This tetrachloro derivative serves as an attractive molecular platform, as the chloro substituent can be easily replaced by a variety of nucleophiles, including other halides (such as Br and I), amines, alcohols, and more.

Tetrachloro laddersiloxane **3** then underwent nucleophilic substitution with sodium azide, using sodium iodide as a catalyst (Scheme 1, **iii**). The target tetraazido laddersiloxane (**4**) was successfully obtained in excellent yield through straightforward extraction and evaporation, without additional purification steps. This compound is noteworthy, as it represents the first tricyclic laddersiloxane with four azido groups. We also attempted similar nucleophilic substitutions with sodium azide on two previously reported tetrachloro-substituted tricyclic 6-8-6 laddersiloxanes [14,16] under comparable conditions (Scheme S1). However, these attempts were unsuccessful in isolating the desired products due to substantial byproduct formation from the cleavage of the ladder skeletons. Even with modification to the reaction conditions, no improvement was observed (Scheme S1). In contrast, during the nucleophilic substitution of compound **3**, the ladder framework remained intact, allowing for easy isolation of pure target compound **4** by simple extraction and evaporation. This contrasting stability between ladder structures previously reported (Scheme S1) and **3** under strong nucleophilic conditions is likely due to the inherent stability differences between tricyclic 6-8-6 and 8-8-8 laddersiloxanes. With identical functional groups, the 8-8-8 laddersiloxanes exhibit higher stability than the 6-8-6 laddersiloxanes [13], as smaller rings in the latter increase both structural rigidity and strain within the ladder framework [20]. Nevertheless, the potential influence of different substituents on the side D-unit Si atoms cannot be completely ruled out.

The ^29^Si NMR spectrum of compound **4** exhibited three singlets at −66.51, −18.58, and 5.11 ppm, assigned, respectively, to the bridged silicon atoms (T-unit Si in blue), the side silicon atoms (D-unit Si in red), and the carbosilanes (Si in pink) (Figure 1b). The chemical shifts of T-unit Si and carbosilanes in tetraazido laddersiloxane **4** align with those previously reported for analogues azido-functionalized T_8_-cages [29], while the D-unit Si are in the same range as reported for 8-8-8 laddersiloxanes [20]. In the ^13^C NMR spectrum of **4** (Figure S5), a singlet at 40.71 ppm is attributed to the methylene protons between the carbosilane and azido group (Si-C*H*_2_-N_3_), showing a downfield shift of approximately 10 ppm compared to the methylene protons between the carbosilane and chloro group (Si-C*H*_2_-Cl) in compound **3**. Furthermore, the infrared spectrum of compound **4** showed a characteristic azido group absorption band at 2093 cm^−1^ (Figure S11). These results confirm that the nucleophilic substitution was complete and that the target compound **4** with azido groups was successfully obtained. ^1^H NMR and ^13^C NMR spectra (Figures S4 and S5) as well as elemental analysis further confirmed the structure of compound **4**. Additionally, the thermogravimetric analysis (TGA) result for compound **4** shows a relatively low Td_5_ (the temperature at which 5% of the initial weigh is lost) of 153 ˚C due to the presence of four unstable azido groups (Table S1, Figure S15).

### 2.2. Synthesis of 8-8-8 Laddersiloxane Ligand and Application in Copper-Catalyzed Oxidative Dehydrogenation of Alcohols

Like related octaazido cubic [29], tetraazido double-decker [38], and tetraazido cyclic silsesquioxanes [39], compound **4** reported here may serve as a versatile building block for designing new hybrid functional materials with applications across various fields.

**Scheme 2:**
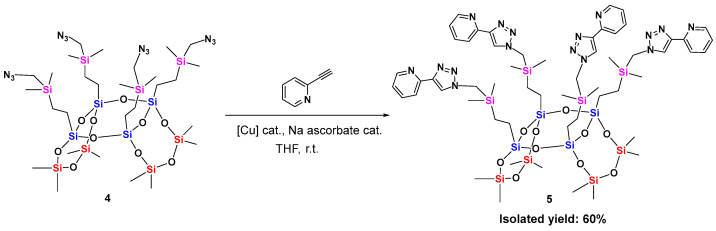
Synthesis of *syn*-type tricyclic 8-8-8 laddersiloxane ligand.

To demonstrate its potential, compound **4** was reacted with the challenging substrate 2-ethynylpyridine through an efficient, quantitative, and atom-economical CuAAC reaction (Scheme 2). Following the reaction conditions used for octaazido cubic T_8_ [29], the reaction was performed at room temperature. The resulting laddersiloxane **5**, bearing four 2-pyridyl-triazole groups on the core, was isolated as an oil in 60% yield after purification by column chromatography. In the ^29^Si NMR spectrum of 2-pyridyltriazole-substituted laddersiloxane **5**, three distinctive signals were observed: −66.68 ppm for the bridged silicon atoms (Si in blue), −18.54 ppm for the side silicon atoms (Si in red), and 4.76 ppm for the carbosilane units (Si in pink) (Figure 1c). Additionally, a singlet at 8.00 ppm in the ^1^H NMR spectrum confirmed the successful formation of the triazole ring (Figure S7). The signal of the methylene groups adjacent to the azido groups, initially at 2.79 ppm in compound **4**, shifted downfield to 3.98 ppm in compound **5**, consistent with the presence of the triazole moiety after cycloaddition (Figures S4 and S7). ^13^C NMR, infrared spectroscopy and MALDI-TOF mass spectrometry further confirmed the structure of compound **5** (Figures S8, S12 and S14). Notably, compound **5** exhibits high thermal stability, with a Td_5_ of 322 °C (Table S1, Figure S16).

Remarkably, the 2-pyridyl-triazole moiety exhibits notable properties as a ligand for transition metals [40,41], either noble (e.g., iridium [42,43], ruthenium [44,45,46,47], palladium [29,48]) or more abundant ones (e.g., nickel [49], iron [50]). The corresponding complexes demonstrate a range of appealing characteristics, such as optical, electronic, biological, sensing, and catalytic properties, making them suitable for applications across diverse fields.

To illustrate the potential of compound **5** bearing four *N,N*- 2-pyridyl-triazole pincer ligands, we turn our attention to the oxidative dehydrogenation of alcohols, a reaction of interest in organic chemistry and biomass valorization [51,52]. For a long time, the oxidation of small organic molecules has represented a significant challenge in synthesis, with issues related to sustainability due to the requirement of toxic reagents and hazardous processes. Fortunately, the development of catalytic methods has helped to mitigate many of these issues. In particular, the combination of transition metal-based catalysts with environmentally benign oxygen from air offers a promising strategy.

Among the various metals, copper stands out for its abundance, cost-effectiveness, and the scalability of copper-catalyzed reactions [53,54]. Additionally, copper-based catalytic systems often employ ligands, especially bi- or tridentate nitrogen ones, which further enhance their efficiency and selectivity [51,55]. For example, Stahl et al. reported a highly efficient benchmark copper catalyst able to promote the oxidative dehydrogenation of a wide range of alcohols [55]. This system combined a copper(I) salt with a bipyridine ligand (bpy/Cu = 1:1) and catalytic amounts of additives, whose roles have been fully determined [56]: 2,2,6,6-tetramethylpiperidine-*N*-oxyl (TEMPO; TEMPO/Cu = 1:1) and N-methylimidazole (NMI; NMI/Cu = 2:1). The authors investigated the mechanism [56] and proposed a seven-step catalytic cycle, with the first step involving the aerobic oxidation of the Cu(I) catalytic species to a Cu(II) complex, resulting in the formation of a dimeric Cu₂O₂ intermediate. Furthermore, this step was found to be strongly influenced by the nature of the ligand and to be rate-determining, especially in the case of benzylic alcohols.

**Scheme 3:**
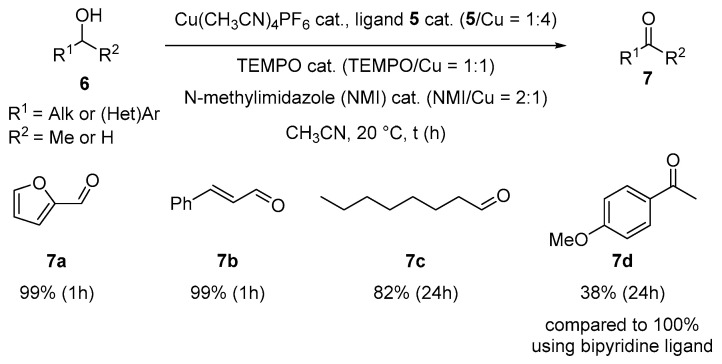
Catalytic activity of the Cu/**5** system in alcohol oxidative dehydrogenation. (Conversions of **6a**–**d** and NMR yields of **7a**–**d** were determined by ^1^H NMR using 1,3,5-trimethoxybenzene as the standard. The selectivity for aldehydes **7a**, **7b** and **7c** is 100%).

In our study, the bipyridine ligand was replaced by the laddersiloxane ligand **5** (2.5 mol%; **5**/Cu = 1:4 using Cu(CH_3_CN)_4_PF_6_ as the copper precursor) (Scheme 3). To our delight, this modified system successfully converted 2-(hydroxymethyl)furan **6a**, a substrate of interest in the context of biomass valorization, into the expected furfural **7a** in a quantitative yield within 1h (Scheme 3). Similarly, cinnamyl alcohol **6b** underwent fast, quantitative and selective oxidation to cinnamaldehyde **7b** within 1h, with the C=C double bond remaining intact (Scheme 3). The more challenging primary aliphatic alcohol **6c**, was also selectively oxidized into 1-octanal **7c** in high yield (82%), although a longer reaction time was required (Scheme 3). Importantly, no carboxylic acids, possibly resulting from over-oxidation, were detected for any of these substrates. We checked that these results were comparable to those obtained with the bipyridine ligand [55]. Interestingly, under the same conditions using **5** as the ligand, 1-(4-methoxyphenyl)ethanol **6d** showed low conversion (38% after 24 h), highlighting the selectivity of the Cu/**5** catalytic system for primary alcohols. Notably, the bipyridine-based system afforded quantitative yields of the corresponding ketone **7d** under identical conditions (Cu(CH_3_CN)_4_PF_6_, 10 mol%; Cu/*N-N*-pincer ratio = 1:1), demonstrating its efficiency in the oxidation of secondary alcohols as effectively as primary ones. This further underscores the added value of ligand **5** in tuning the selectivity of the catalytic system towards primary alcohols.

## 3. Materials and Methods

### 3.1. General Considerations

All reactions were performed under an argon atmosphere using the standard Schlenk technique, except where otherwise noted. Tetrahydrofuran (THF, InChI = 1S/C4H8O/c1-2-4-5-3-1/h1-4H2), toluene (InChI = 1S/C7H8/c1-7-5-3-2-4-6-7/h2-6H,1H3), and N,N-dimethylformamide (DMF, InChI = 1/C3H7NO/c1-3(5)4-2/h1-2H3,(H,4,5)) were dried using the mBRAUN purification system. Triethylamine was distilled from potassium hydroxide (InChI = 1S/K.H2O/h;1H2/q+1;/p-1), stored on potassium hydroxide under an argon atmosphere with protection from light. 1,3-dichloro-1,1,3,3-tetramethylsiloxane (InChI = 1S/C4H12Cl2OSi2/c1-8(2,5)7-9(3,4)6/h1-4H3), sodium azide (InChI = 1S/N3.Na/c1-3-2;/q-1;+1), sodium L-ascorbate (InChI = 1S/C6H8O6.Na/c7-1-2(8)5-3(9)4(10)6(11)12-5;/h2,5,7-10H, 1H2;/q;+1/p-1/t2-,5+;/m0./s1), and 2-ethynylpyridine (InChI = 1S/C7H5N/c1-2-7-5-3-4-6-8-7/h1,3-6H) were purchased from Tokyo Chemical Industry (TCI) Co., Ltd. (Tokyo, Japan); (Chloromethyl)dimethylsilane (InChI = 1S/C3H9ClSi/c1-5(2)3-4/h5H,3H2,1-2H3) and Karstedt’s catalyst (in xylene, 2% Pt, InChI = 1S/C8H18OSi2.Pt/c1-7-10(3,4)9-11(5,6)8-2;/h7-8H,1-2H2,3-6H3) was purchased from Sigma-Aldrich (St. Louis, MO, USA); copper(II) sulfate pentahydrate (InChI = 1S/Cu.H2O4S/c;1-5(2,3)4/h;(H2,1,2,3,4)/q+2;/p-2), sodium iodide (InChI = 1S/HI.Na/h1H;/q;+1/p-1) were purchased from FUJIFILM Wako Pure Chemical Co. Chemical Reagents (Osaka, Japan). All of these reagents were used as received, without further purification. Caution: Sodium azide (NaN_3_) is highly toxic and poses a potential explosion hazard. Therefore, it is essential to take appropriate safety precautions when conducting the azidation reaction reported in this work.

*For characterizing compounds* **3**–**5**: The Fourier transformation nuclear magnetic resonance (NMR) spectra were obtained using JEOL JNM-ECA 600 (^1^H at 600.17 MHz, ^13^C at 150.91 MHz, ^29^Si at 119.24 MHz) NMR instrument (JEOL Ltd., Akishima, Tokyo, Japan). For ^1^H NMR, chemical shifts are reported as δ units (ppm) relative to SiMe_4_ (TMS) and the residual solvents peaks were used as standards. For ^13^C NMR and ^29^Si NMR, chemical shifts are reported as δ units (ppm) relative to SiMe_4_ (TMS), the residual solvents peaks were used as standards and spectra were obtained with complete proton decoupling. MALDI-TOF (matrix-assisted laser desorption/ionization coupled time-of-flight) mass analyses were carried out with a Shimadzu AXIMA^®^ instrument (Shimadzu Corporation, Kyoto, Japan) using 2,5-dihydroxybenzoic acid (dithranol) as the matrix and AgNO_3_ as the ion source. All reagents used were of analytical grade. Element analyses were performed by the Center for Material Research by Instrumental Analysis (CIA), Gunma University, Japan. IR spectra were measured with a Shimadzu IRSpirit FTIR (Shimadzu Corporation, Kyoto, Japan). TGA was carried out with a Rigaku (Tokyo, Japan) thermogravimetric analyzer (Thermoplus TG-8120). The investigations were carried out under a nitrogen flow (250 mL min^−1^) or an air flow (300 mL min^−1^) with a heating rate of 10 ˚C min^−1^. All samples were measured with temperatures ranging from 50 to 1000 ˚C, where they remained for 5 min. The weight loss and heating rate were continuously recorded throughout the experiment.

*For catalytic reactions:* Cu(CH_3_CN)_4_PF_6_ (InChI = 1S/4C2H3N.Cu.F6P/c4*1-2-3;;1-7(2,3,4,5)6/h4*1H3;;/q;;;;+1;-1), TEMPO (InChI = 1S/C9H18NO/c1-8(2)6-5-7-9(3,4)10(8)11/h5-7H2,1-4H3), N-methylimidazole (InChI = 1S/C4H6N2/c1-6-3-2-5-4-6/h2-4H,1H3), alcohols **6a**–**d** (**6a**: InChI = 1S/C5H6O2/c6-4-5-2-1-3-7-5/h1-3,6H,4H2; **6b**: InChI = 1S/C9H10O/c10-8-4-7-9-5-2-1-3-6-9/h1-7,10H,8H2/b7-4+; **6c**: InChI = 1S/C8H18O/c1-2-3-4-5-6-7-8-9/h9H,2-8H2,1H3; **6d**: InChI = 1S/C9H12O2/c1-11-9-4-2-8(3-5-9)6-7-10/h2-5,10H,6-7H2,1H3) and acetonitrile (InChI = 1S/C2H3N/c1-2-3/h1H3) were purchased from Merck (Darmstadt, Germany) or TCI Chemicals, and 1,3,5-trimethoxybenzene was purchased from Alfa Aesar Co., Ltd. (Ward Hill, MA, USA). All reagents were used as received without further purification. Liquid ^1^H NMR spectra were recorded on a Bruker Avance 400 MHz spectrometer in CDCl_3_ at room temperature. For ^1^H NMR, chemical shifts were reported as δ units (ppm) relative to SiMe_4_ (TMS), and the residual solvent peaks were used as standards.

### 3.2. Experimental Procedures and Characterization Data for Synthetic Compounds ***2***–***5***

Synthesis of (1*R*,3*S*,9*R*,11*S*)-5,5,7,7,13,13,15,15-octamethyl-1,3,9,11-tetravinyl-2,4,6,8,10,12,14,16,17,18-decaoxa-1,3,5,7,9,11,13,15-octasilatricyclo[9.5.1.13,9]octadecane (**2**)

An argon-purged, three-necked round-bottom flask equipped with a stir bar and an addition-funnel was charged with all-*cis*-tetravinylcyclotetrasiloxanolate ([ViSi(OK)O]_4_ (**1**), 0.5 g, 0.99 mmol), which had been preliminarily dried at 60 °C for 2 h, along with distilled triethylamine (0.58 mL, 4.2 mmol) and anhydrous THF (20 mL). 1,3-dichloro-1,1,3,3-tetramethyldisiloxane (0.41 mL, 2.1 mmol) and anhydrous THF (30 mL) were added to the addition-funnel under an argon atmosphere. This solution was then slowly added dropwise to the flask at 0 °C over 1.5 h. After the addition, the reaction mixture was stirred at 25 °C for an additional hour. A saturated NH_4_Cl solution was then added to neutralize the triethylamine, followed by CHCl_3_ to extract the product. The organic layer was washed three times with brine, dried over anhydrous Na_2_SO_4_, and concentrated on a rotary evaporator to obtain the desired product **3** (0.35 g, 58%) as a white solid without further purification. The analytical data matched those previously reported [20].

Synthesis of (1*R*,3*S*,9*R*,11*S*)-1,3,9,11-tetrakis(2-((chloromethyl)dimethylsilyl)ethyl)-5,5,7,7,13,13,15,15-octamethyl-2,4,6,8,10,12,14,16,17,18-decaoxa-1,3,5,7,9,11,13,15-octasilatricyclo[9.5.1.13,9]octadecane (**3**)

After standard cycles of evacuation and backfilling with dry argon, an oven-dried Schlenk flask equipped with a magnetic stir bar was charged with compound **2** (0.3 g, 0.49 mmol), (chloromethyl)dimethylsilane (0.36 mL, 3.0 mmol), and dry toluene (2.5 mL). Karstedt’s catalyst (2% Pt, commercial bottle, diluted 100 times in toluene under argon, 560 μL, 0.49 μmol, 0.1 mol%) was then added. The reaction mixture was heated to 40 °C and stirred at this temperature for 19 h. After completion, the mixture was passed through a short silica plug, which was washed three times with diethyl ether, and the filtrate was collected and evaporated on a rotary evaporator to obtain the desired product **3** (0.51 g, 99%) as a brown viscous oil without further purification.

**^1^H NMR (600.17 MHz, CDCl_3_)**: δ = 0.10 (s, 24H), 0.11 (s, 12H), 0.15 (s, 12H), 0.48–0.53 (m, 8H), 0.63–0.68 (m, 8H), 2.79 (s, 8H) ppm.
**^13^C{^1^H} NMR (150.91 MHz, CDCl_3_)**: δ = −4.96, 0.79, 0.89, 5.23, 5.30, 30.02 ppm.
**^29^Si{^1^H} NMR (119.24 MHz, CDCl_3_)**: δ = −66.39, −18.66, 5.28 ppm.
**MALDI-TOF MS (*m*/*z*)**: 1068.88 ([M+Na]^+^, calcd. 1069.10).
**Elemental analysis**: Calcd for C_28_H_72_Cl_4_O_10_Si_12_: C, 32.10; H, 6.93; Found: C, 31.70; H, 7.16.

Synthesis of (1*R*,3*S*,9*R*,11*S*)-1,3,9,11-tetrakis(2-((azidomethyl)dimethylsilyl)ethyl)-5,5,7,7,13,13,15,15-octamethyl-2,4,6,8,10,12,14,16,17,18-decaoxa-1,3,5,7,9,11,13,15-octasilatricyclo[9.5.1.13,9]octadecane (**4**)

After standard cycles of evacuation and backfilling with dry argon, an oven-dried Schlenk flask equipped with a magnetic stir bar was charged with compound **3** (0.26 g, 0.25 mmol), NaI (0.02 g, 0.125 mmol), and NaN_3_ (0.19 g, 3 mmol). Dry THF (7 mL) and dry DMF (17 mL) were then added to the mixture at room temperature under an argon atmosphere with vigorous stirring. The reaction mixture was heated to 60 °C and stirred at 60 °C for 24 h. After cooling to room temperature, dichloromethane was added slowly, and the mixture was stirred for approximately 10 min. Ice was then added, and the mixture was stirred for an additional 30 min. The organic layer was separated, washed twice with brine, dried over anhydrous Na_2_SO_4_, and concentrated on a rotary evaporator to obtain the desired product **4** (0.25 g, 90%) as a dark yellow viscous oil without further purification.

**^1^H NMR (600.17 MHz, CDCl_3_)**: δ = 0.10 (s, 24H), 0.11 (s, 12H), 0.15 (s, 12H), 0.48–0.52 (m, 8H), 0.60–0.64 (m, 8H), 2.79 (s, 8H) ppm.
**^13^C{^1^H} NMR (150.91 MHz, CDCl_3_)**: δ = −4.62, 0.78, 0.89, 5.24, 5.79, 40.71 ppm.
**^29^Si{^1^H} NMR (119.24 MHz, CDCl_3_)**: δ = −66.51, −18.58, 5.11 ppm.
**Elemental analysis**: Calcd for C_28_H_72_N_12_O_10_Si_12_: C, 31.31; H, 6.76; N, 15.65; Found: C, 31.59; H, 7.03; N, 13.84.

Synthesis of 2,2′,2″,2″′-((((((1*R*,3*S*,9*R*,11*S*)-5,5,7,7,13,13,15,15-octamethyl-2,4,6,8,10,12,14,16,17,18-decaoxa-1,3,5,7,9,11,13,15-octasilatricyclo[9.5.1.13,9]octadecane-1,3,9,11-tetrayl)tetrakis(ethane-2,1-diyl))tetrakis(dimethylsilanediyl))tetrakis(methylene))tetrakis(1*H*-1,2,3-triazole-1,4-diyl))tetrapyridine (**5**)

After standard cycles of evacuation and backfilling with dry argon, an oven-dried Schlenk flask equipped with a magnetic stir bar was charged with compound **4** (0.07 g, 0.065 mmol), 2-ethynylpyridine (39 μL, 0.39 mmol), and dry THF (4.3 mL). To this solution, 12 μL of degassed copper sulfate pentahydrate aqueous solution (125 mg/mL, 0.0065 mmol) and sodium ascorbate (0.0064 g, 0.033 mmol) were added under argon. The mixture was stirred at 25 °C for 1 day. Subsequently, an additional 12 μL of degassed copper sulfate pentahydrate aqueous solution (125 mg/mL, 0.0065 mmol) and sodium ascorbate (0.0064 g, 0.033 mmol) were added under argon, and the reaction mixture was allowed to stir for another day at 25 °C. A third addition of 12 μL of degassed copper sulfate pentahydrate aqueous solution (125 mg/mL, 0.0065 mmol) and sodium ascorbate (0.0064 g, 0.033 mmol) was made, followed by stirring for an additional day at 25 °C. Water was then added, and the reaction mixture was extracted three times with dichloromethane. The combined organic layers were washed three times with brine, dried over anhydrous Na_2_SO_4_, and concentrated on a rotary evaporator to yield a crude product. This crude product was dried under vacuum at 100 °C for 3 h to remove excess 2-ethylnylpyridine. Finally, the crude product was purified by column chromatography to remove copper (eluent: CH_2_Cl_2_/MeOH = 10/1), obtaining the desired product **5** (0.058 g, 60%) as a dark yellow viscous oil.

**^1^H NMR (600.17 MHz, CDCl_3_)**: δ = 0.05 (s, 12 H), 0.11 (s, 36H), 0.52–0.55 (m, 8H), 0.68–0.71 (m, 8H), 3.98 (s, 8H), 7.14–7.16 (m, 4H), 7.69–7.70 (m, 4H), 8.00 (s, 4H), 8.11 (d, *J* = 7.9 Hz, 4H), 8.52 (d, *J* = 4.1 Hz, 4H) ppm.
**^13^C{^1^H} NMR (150.91 MHz, CDCl_3_)**: δ = −4.43, 0.74, 0.89, 5.14, 5.91, 40.75, 120.17, 122.72, 122.88, 136.92, 148.17, 149.39, 150.66 ppm.
**^29^Si{^1^H} NMR (119.24 MHz, CDCl_3_)**: δ = −66.68, −18.54, 4.76 ppm.
**MALDI-TOF MS (*m*/*z*)**: 1507.48 ([M+Na]^+^, calcd. 1507.43); 1523.40 ([M+K]^+^, calcd. 1523.41).
**Elemental analysis**: Calcd for C_56_H_92_N_16_O_10_Si_12_: C, 45.25; H, 6.24; N, 15.08; Found: C, 45.45; H, 6.63; N, 14.10.

### 3.3. Experimental Procedure for Catalytic Reactions

In a culture tube equipped with a stirrer bar, a solution of **5** (0.0125 mmol, 18.6 mg) in acetonitrile (0.25 mL) was added to a solution of Cu(CH_3_CN)_4_PF_6_ (0.05 mmol, 18.6 mg) in acetonitrile (0.25 mL), and the mixture was stirred for 5 min at room temperature. Next, solutions of TEMPO (0.05 mmol, 7.8 mg in 0.25 mL of CH_3_CN) and 1-methylimidazole NMI (0.5 mL of 0.2 M solution in CH_3_CN) were introduced. Finally, the alcohol (0.5 mmol, 43 µL for **6a**, 67,1 mg for **6b**, 78 µL for **6c**, 76 mg for **6d**) and 0.25 mL of CH_3_CN were added. The resulting mixture was stirred at 20 °C in an open-air atmosphere for a duration depending on the substrate (Scheme 3).

Next, the NMR standard 1,3,5-trimethoxybenzene (0.1 mmol, 16.8 mg) was added and after the addition of dichloromethane (1 mL); the reaction mixture was filtered through a plug of Celite and evaporated to dryness. The resulting crude product was dissolved in CDCl_3_ and analyzed by ^1^H NMR to determine the conversions of **6a**–**d** and the yields of **7a**–**d**.

## 4. Conclusions

In this study, we successfully synthesized a novel *syn*-type tricyclic 8-8-8 laddersiloxane bearing four azido groups, using a highly efficient and selective approach involving hydrosilylation and nucleophilic substitution (92% yield over the two steps). The versatility of the azido functional groups makes this laddersiloxane a promising precursor for the fabrication of a wide range of hybrid materials. To illustrate its potential, the azido-substituted laddersiloxane was converted via click chemistry into a multidentate ligand with four 2-triazole-pyridyl moieties. The latter was employed in the copper-catalyzed oxidative dehydrogenation of alcohols. The resulting catalytic system exhibited excellent activity and remarkable selectivity for primary alcohols, thereby highlighting the promising potential of this novel laddersiloxane in catalysis. This work opens new pathways for further utilization of laddersiloxane frameworks in catalysis and other advanced material applications.

## Data Availability

All data and material described in this work are available in this article or in the Supplementary Materials.

## Supplementary Materials

The following supporting information can be downloaded at: https://www.mdpi.com/article/10.3390/molecules30020373/s1, ^1^H, ^13^C, ^29^Si NMR spectra for compounds **3**–**5**; structures of the previously reported chloro-functionalized 6-8-6 laddersiloxanes; infrared spectra for compounds **3**–**5**; MALDI-TOF mass spectra for compounds **3** and **5**; thermogravimetry/differential thermal analysis (TG/DTA) spectra for compounds **4** and **5**. Figure S1: ^1^H NMR spectrum for compound **3**; Scheme S1: Nucleophilic substitution of the previously reported tetrachloro-substituted *syn*-type tricyclic 6-8-6 laddersiloxanes (compounds **a** and **b**). Table S1: Thermal properties for compounds **4** and **5** under N_2_; Figure S2: ^13^C NMR spectrum for compound **3**; Figure S3: ^29^Si NMR spectrum for compound **3**; Figure S4: ^1^H NMR spectrum for compound **4**; Figure S5: ^13^C NMR spectrum for compound **4**; Figure S6: ^29^Si NMR spectrum for compound **4**; Figure S7: ^1^H NMR spectrum for compound **5**; Figure S8: ^13^C NMR spectrum for compound **5**; Figure S9: ^29^Si NMR spectrum for compound **5**; Figure S10: Infrared spectrum for compound **3**; Figure S11: Infrared spectrum for compound **4**; Figure S12: Infrared spectrum for compound **5**; Figure S13: MALDI-TOF analysis for compound **3**; Figure S14: MALDI-TOF analysis for compound **5**; Figure S15: Thermogravimetric graphic of compound **4**; Figure S16: Thermogravimetric graphic of compound **5**.
