# Supplementary material for: Synthesis of an Azido-Substituted 8-Membered Ring Laddersiloxane and Its Application in Catalysis
Source: Molecules. 2025 Jan 17;30(2):373. doi: 10.3390/molecules30020373 (PMC11767378; doi:10.3390/molecules30020373)
Supplement: Supplementary file 1 [file molecules-30-00373-s001.zip › molecules-3407726-supplementary.pdf]

Synthesis of an Azido-Substituted 8-Membered Ring Laddersiloxane and Its Application in Catalysis

Yujia Liu [1,*], Niyaz Yagafarov [1], Koki Shimamura [1,2], Nobuhiro Takeda [1], Masafumi Unno [1,*] and Armelle Ouali [2,*]

[1] Department of Chemistry and Chemical Biology, Graduate School of Science and Technology, Gunma University, 1-5-1 Tenjin-cho, Kiryu 376-8515, Japan; driagafarov@gunma-u.ac.jp (N.Y.); t241a052@gunma-u.ac.jp (K.S.); ntakeda@gunma-u.ac.jp (N.T.)

[2] ICGM, Univ Montpellier, CNRS, ENSCM (Institut Charles Gerhardt Montpellier, Université de Montpellier, Centre National de la Recherche Scientifique, École Nationale Supérieure de Chimie de Montpellier), 1919 Route de Mende, CEDEX 05, 34293 Montpellier, France

* Correspondence: yliu@gunma-u.ac.jp (Y.L.); unno@gunma-u.ac.jp (M.U.); armelle.ouali@enscm.fr (A.O.); Tel.: +81-27-730-1234 (Y.L. & M.U.); +33-44-879-2010 (A.O.)

Supporting information

Table of contents **Pages**

1. ^{1}H, ^{13}C and ^{29}Si NMR spectra for compounds **3-5** S2
2. Structures of the previously reported chloro-functionalized 6-8-6 laddersiloxanes S7
3. Infrared spectra for compounds **3-5** S8
4. MALDI-TOF MS (m/z) for compounds **3** and **5** S9
5. Thermogravimetric analysis for compounds **4** and **5** S10

1. ^{1}H, ^{13}C, and ^{29}Si spectra for compounds 3-5

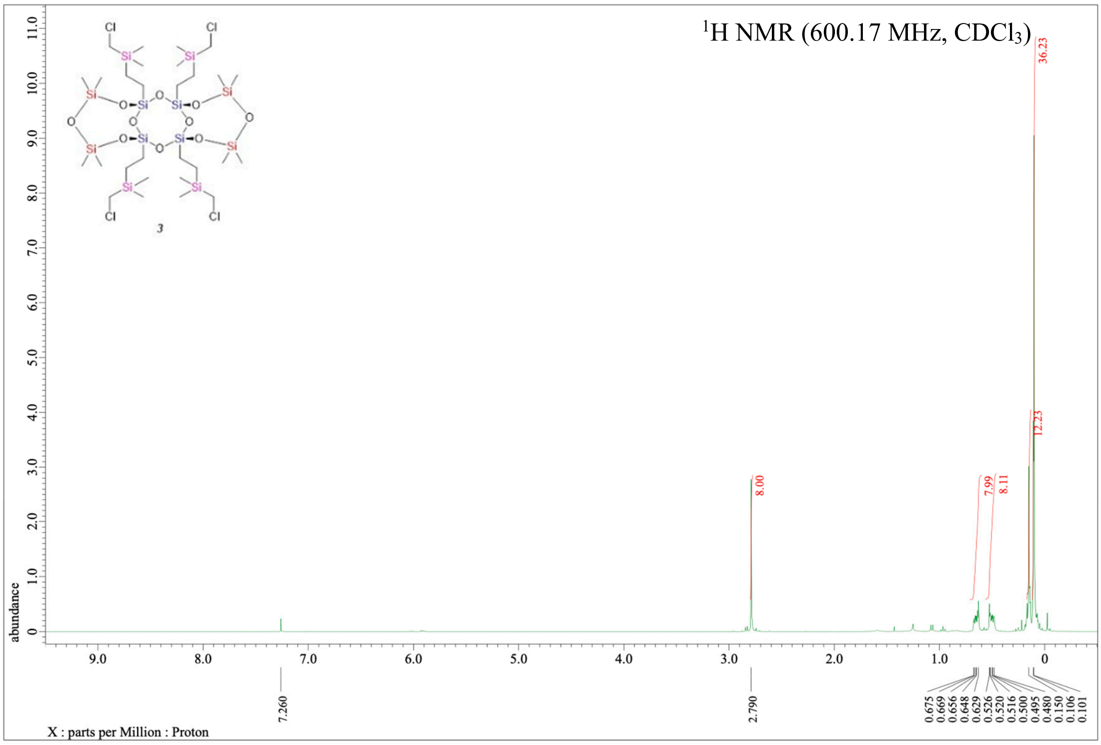

Figure S1: ^{1}H NMR spectrum for compound **3**

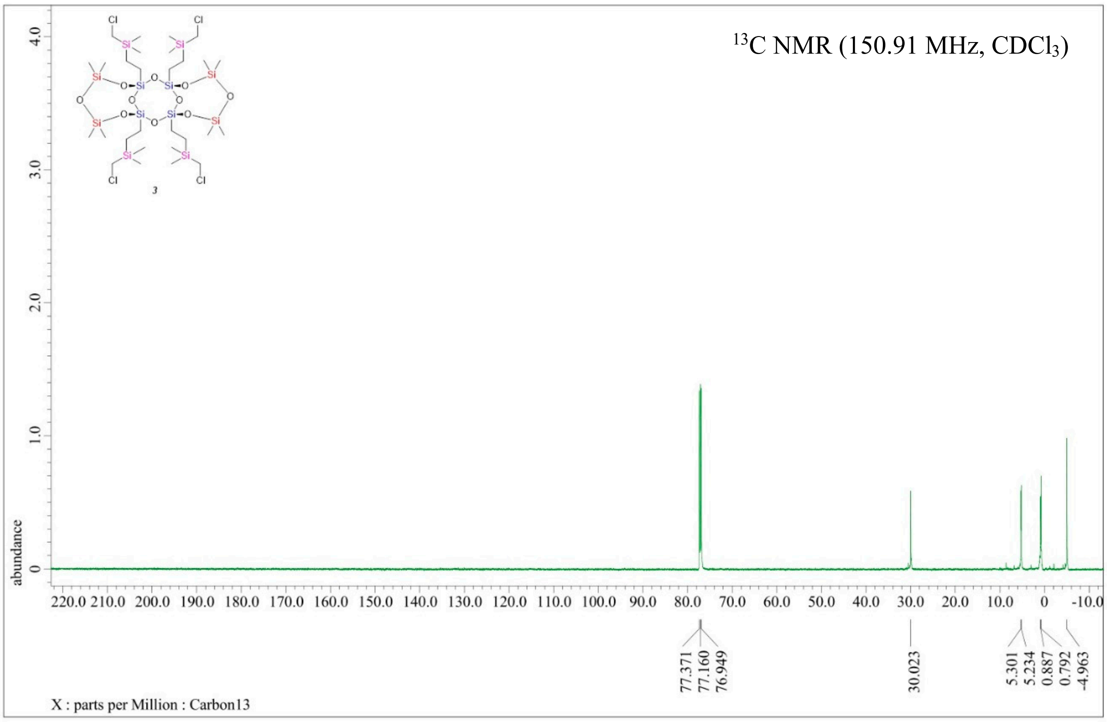

Figure S2: ^{13}C NMR spectrum for compound **3**

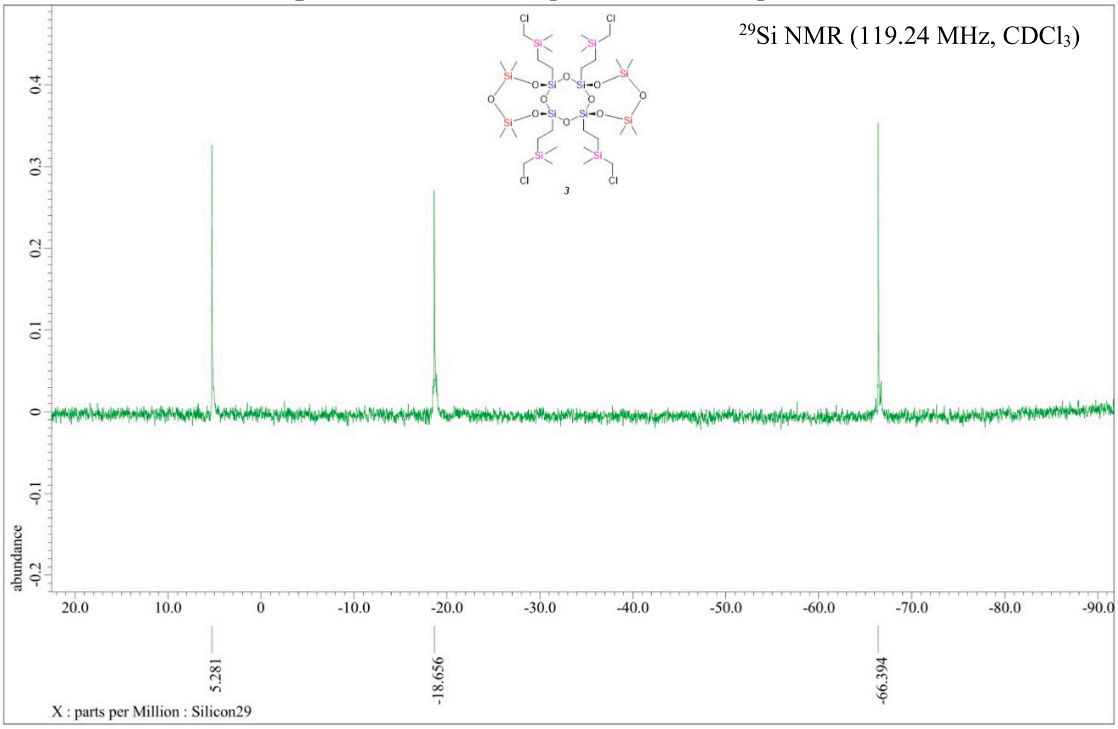

Figure S3: ^{29}Si NMR spectrum for compound **3**

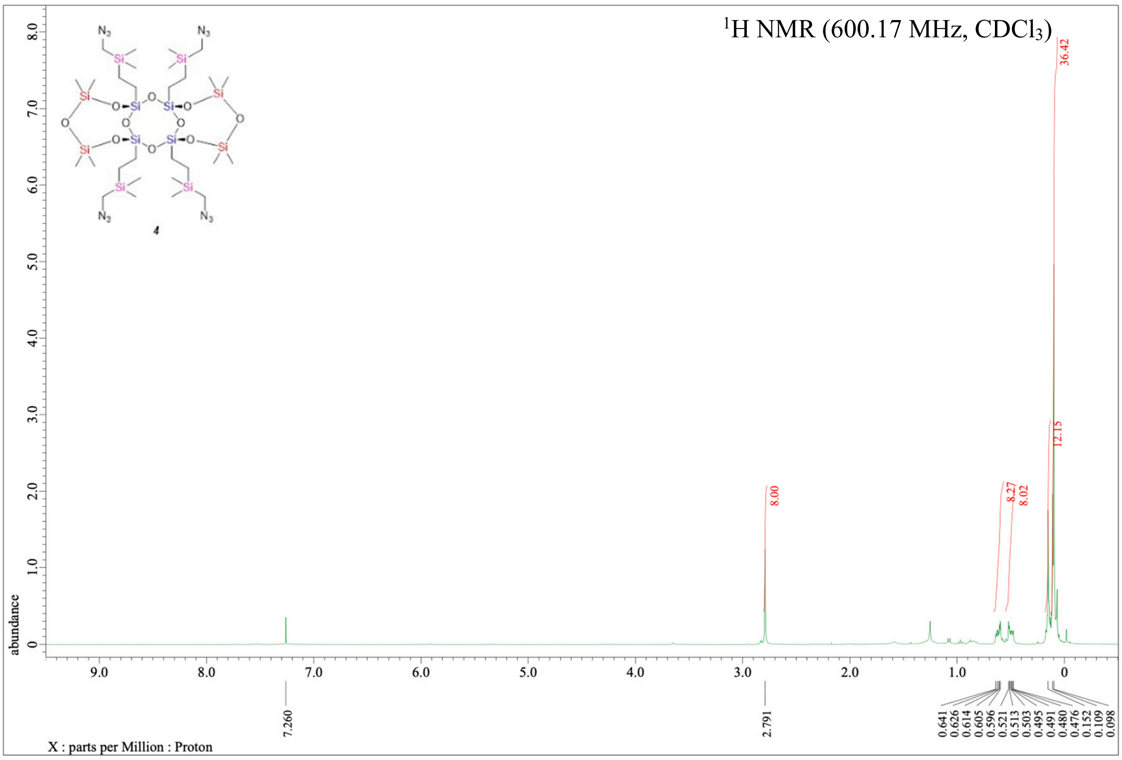

Figure S4: ^{1}H NMR spectrum for compound **4**

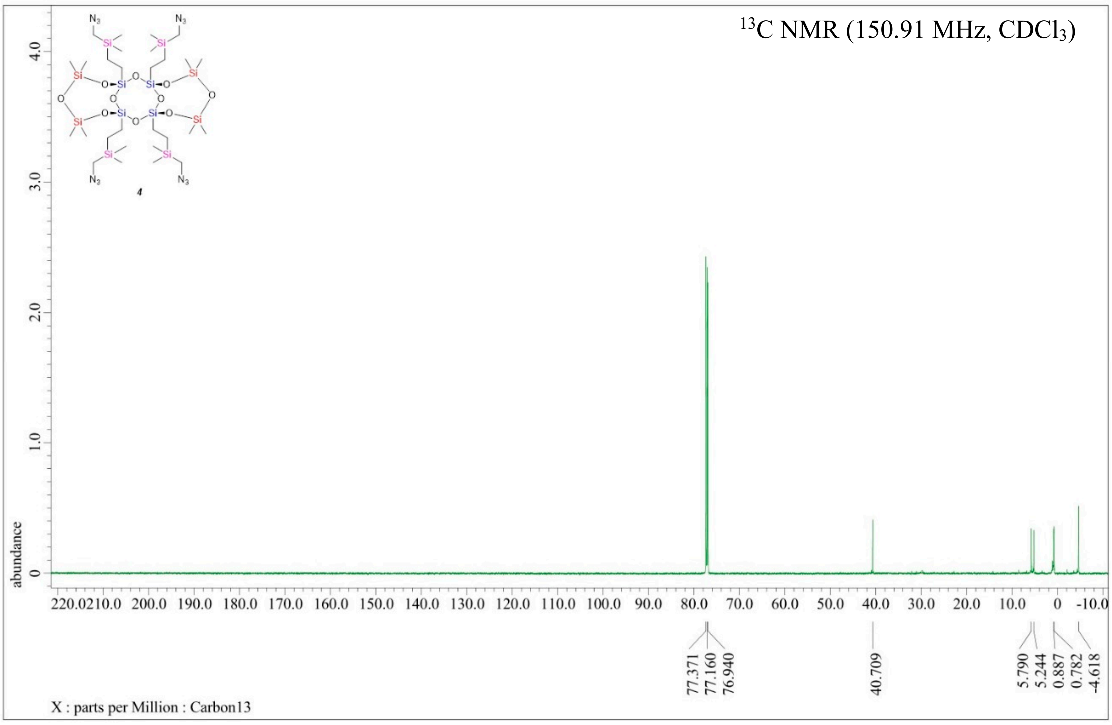

Figure S5: ^{13}C NMR spectrum for compound **4**

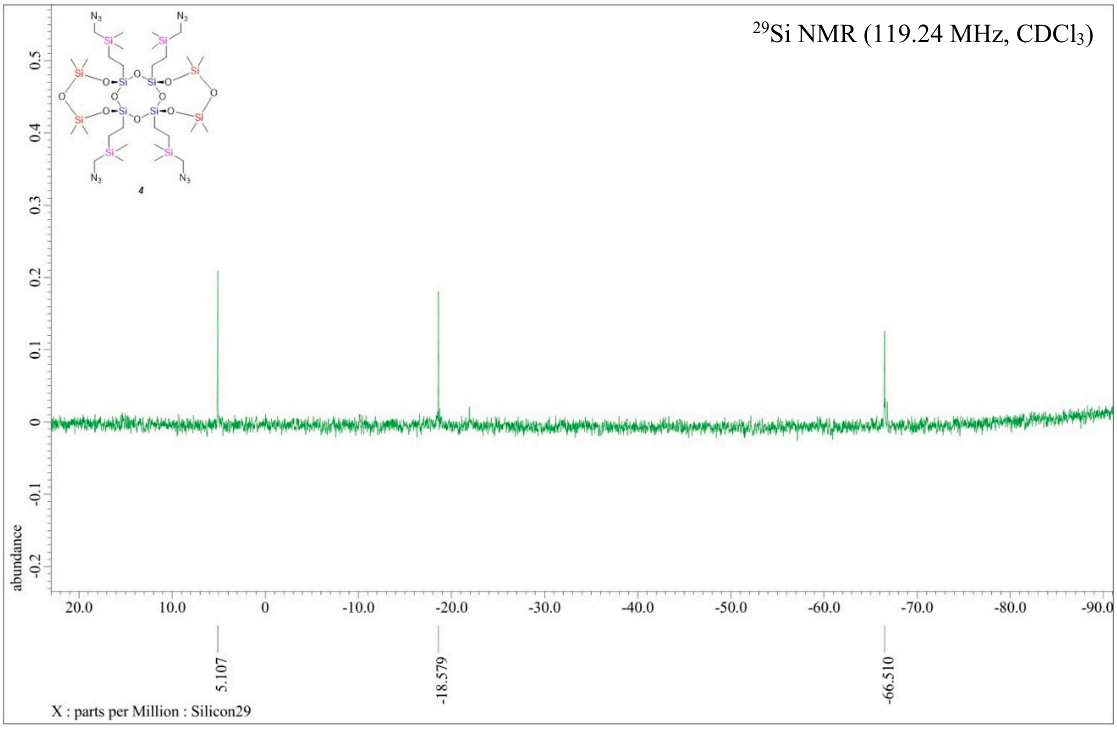

Figure S6: ^{29}Si NMR spectrum for compound **4**

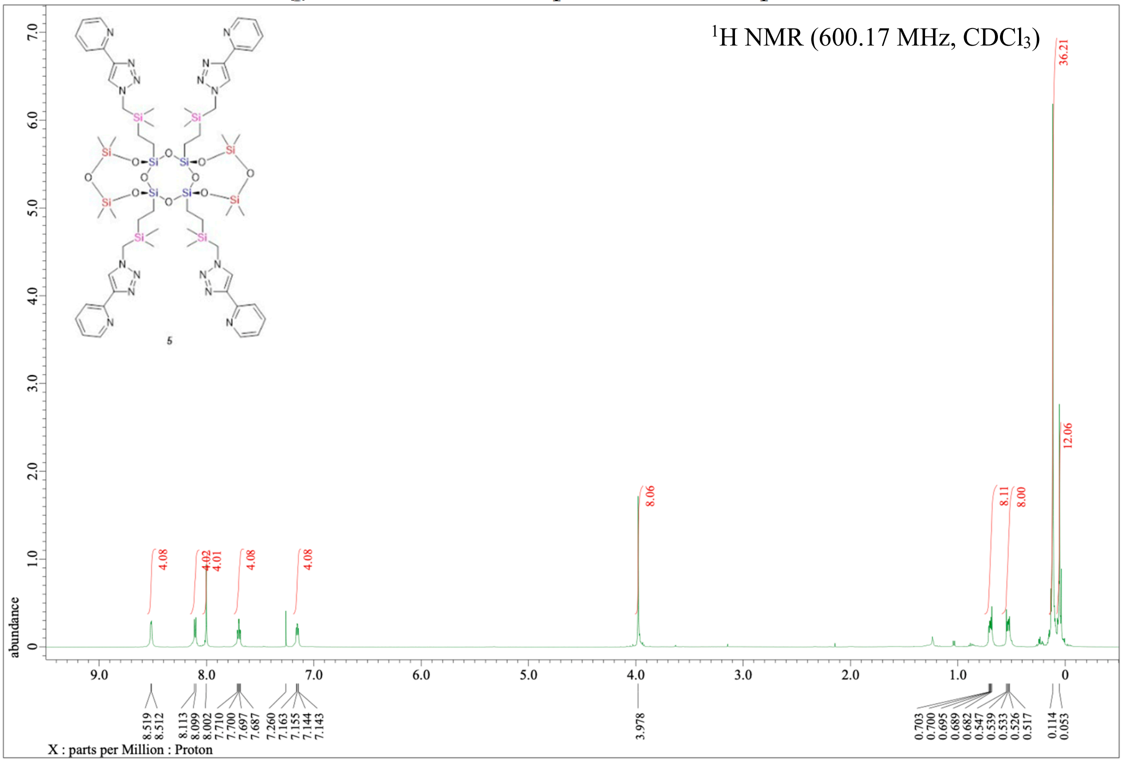

Figure S7: ^{1}H NMR spectrum for compound **5**

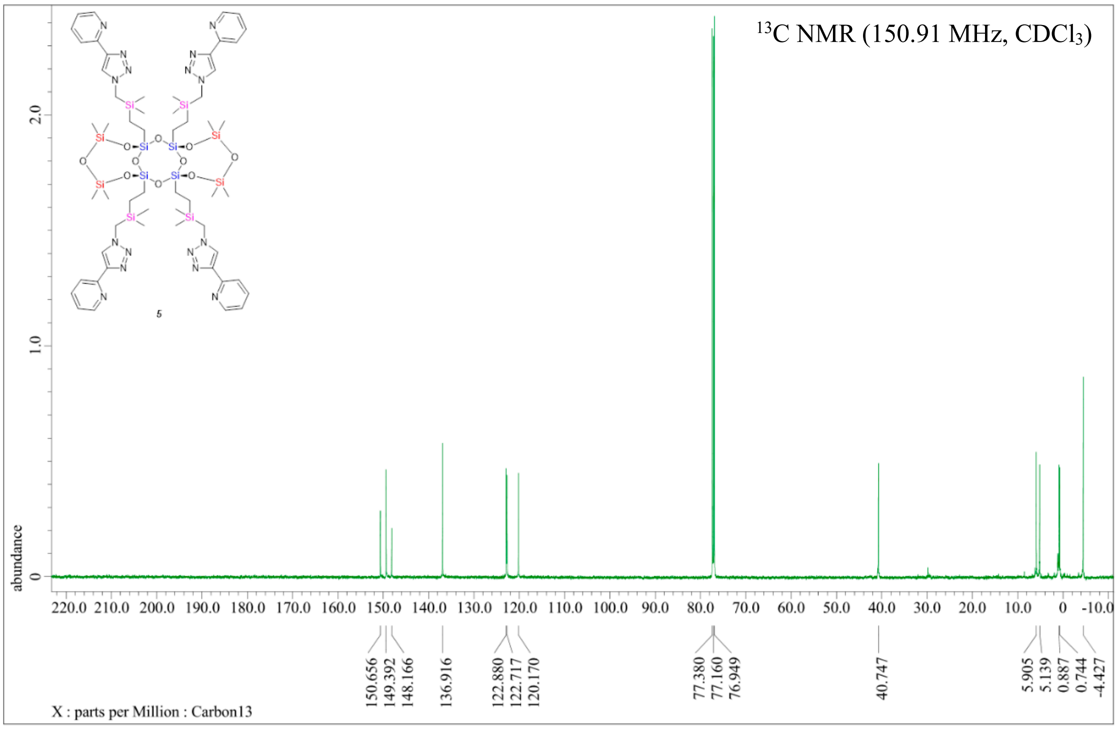

Figure S8: ^{13}C NMR spectrum for compound **5**

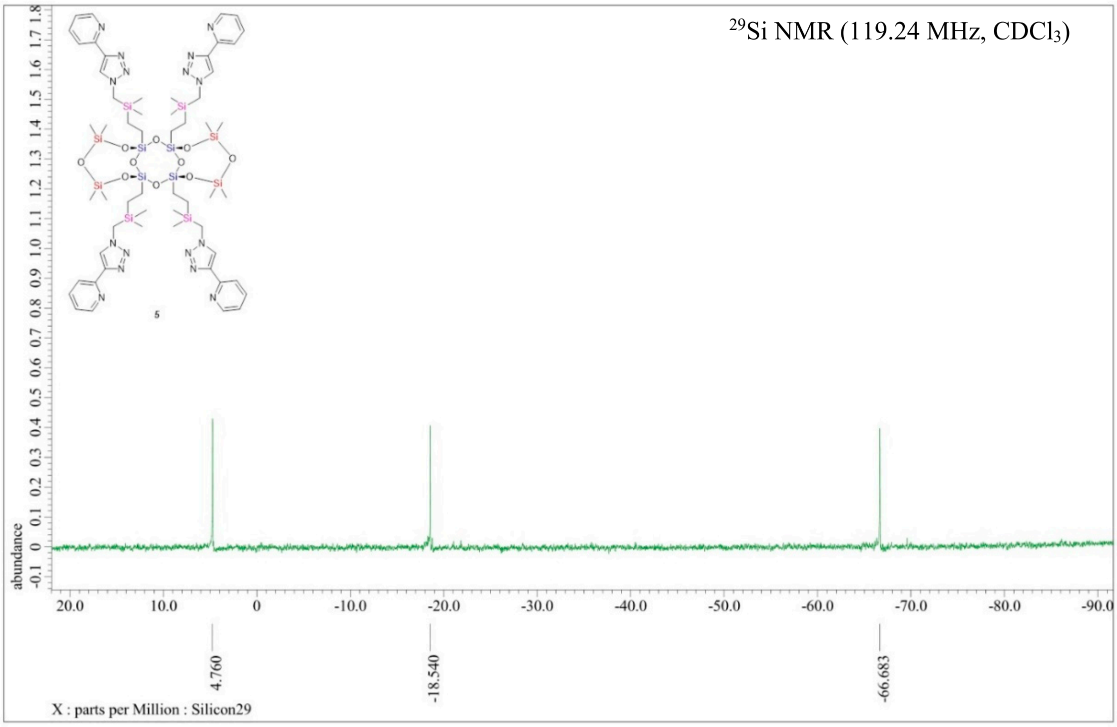

Figure S9: ^{29}Si NMR spectrum for compound **5**

2. Structures of the previously reported chloro-functionalized 6-8-6 laddersiloxanes

Attempts to convert the chloro 6-8-6 laddersiloxanes **a**[1] and **b**[2] (Scheme S1) into the corresponding azido compounds under the conditions described in Scheme S1 were unsuccessful. Under similar conditions (using NaN_3, NaI, DMF, at 60 ˚C), the ladder structures were degraded. Modifications such as changing the solvent (methyl isobutyl ketone, acetylacetone, diethyl carbonate, ethyl acetoacetate, toluene, acetonitrile, ethyl acetate, dimethyl sulfoxide, 2-butanone), altering the reaction temperature (20 to 100 °C), or adding 18-crown-ether or sodium iodide (NaI), did not improve the performances. The results varied: either the starting materials were quantitatively recovered (in the absence of NaI), or iodo-substituted derivatives were observed (in the presence of NaI), or the ladder scaffolds were damaged.

[1] Liu, Y.; Onodera, K.; Takeda, N.; Ouali, A.; Unno, M. Synthesis and Characterization of Functionalizable Silsesquioxanes with Ladder-type Structures. *Organometallics* **2019**, *38*, 4373-4376.

[2] Liu, Y.; Katano, M.; Yingsukkamol, P.; Takeda, N.; Unno, M.; Ouali, A. Tricyclic 6-8-6 laddersiloxanes derived from all-*cis*-tetravinylcyclotetrasiloxanolate: Synthesis, characterization and reactivity. *J. Organomet. Chem.* **2022**, *959*, 122213.

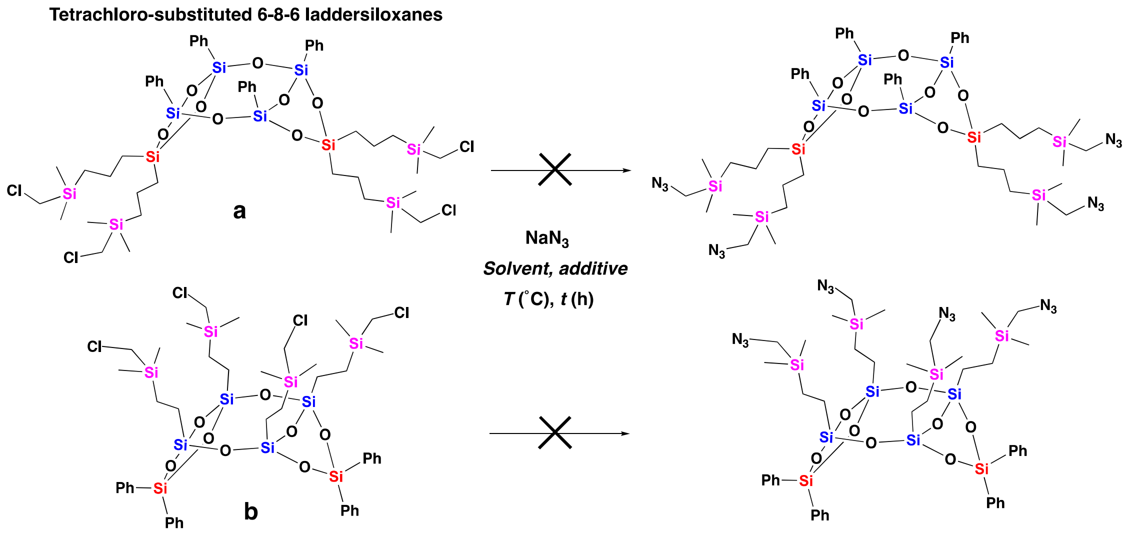

Scheme S1. Nucleophilic substitution of the previously reported tetrachloro-substituted *syn*-type tricyclic 6-8-6 laddersiloxanes (compounds **a** and **b**)[1,2].

3. Infrared spectra for compounds 3-5

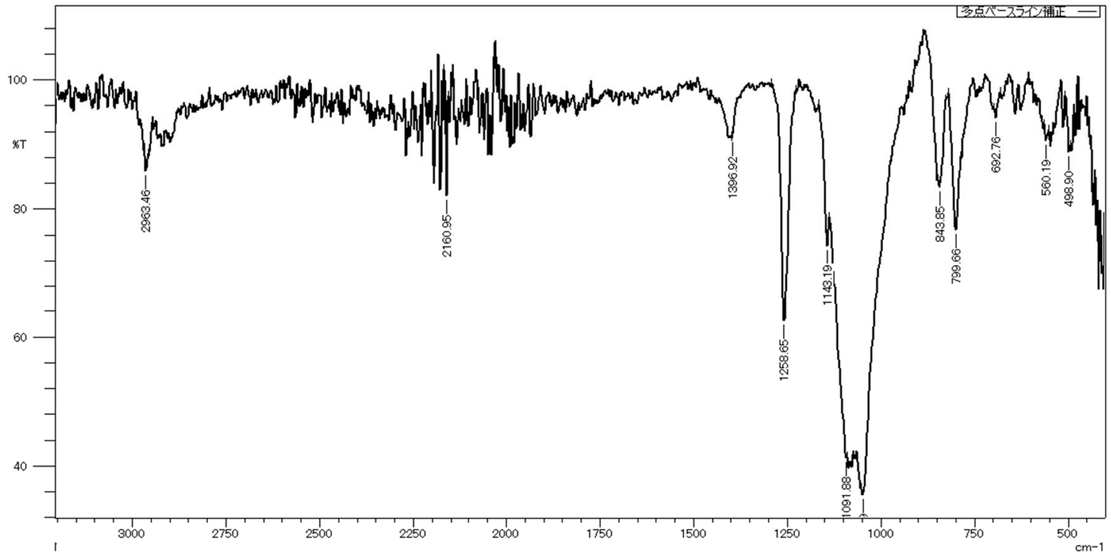

Figure S10: Infrared spectrum for compound **3**

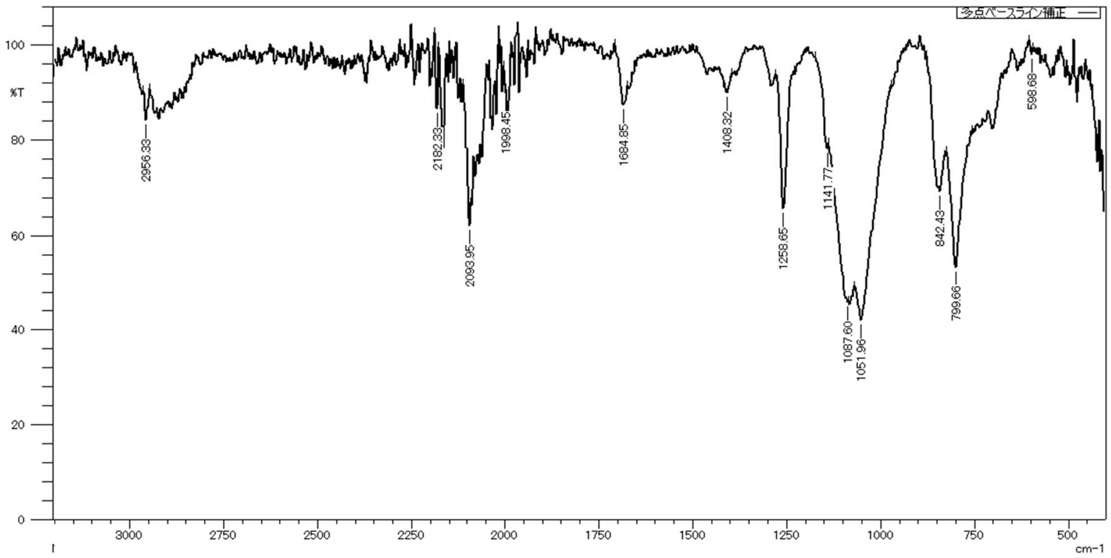

Figure S11: Infrared spectrum for compound **4**

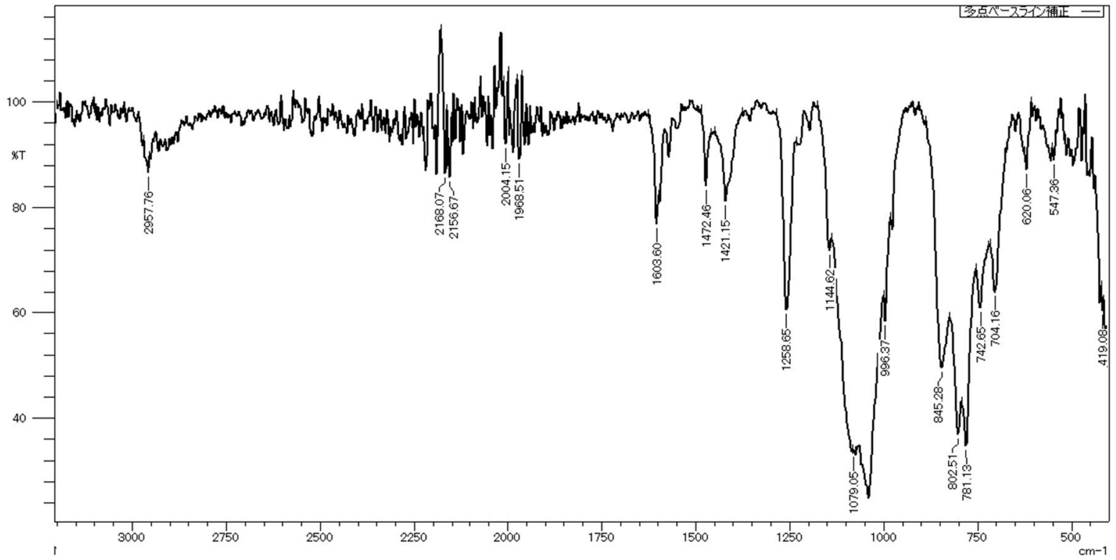

Figure S12: Infrared spectrum for compound **5**

4. MALDI-TOF MS (m/z) for compounds 3 and 5

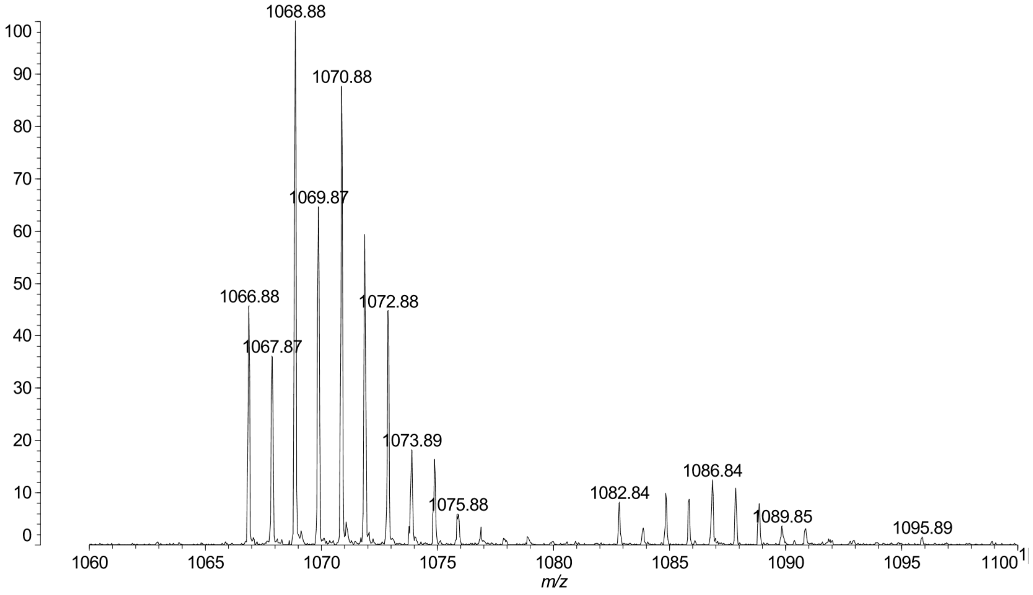

Figure S13: MALDI-TOF analysis for compound **3**

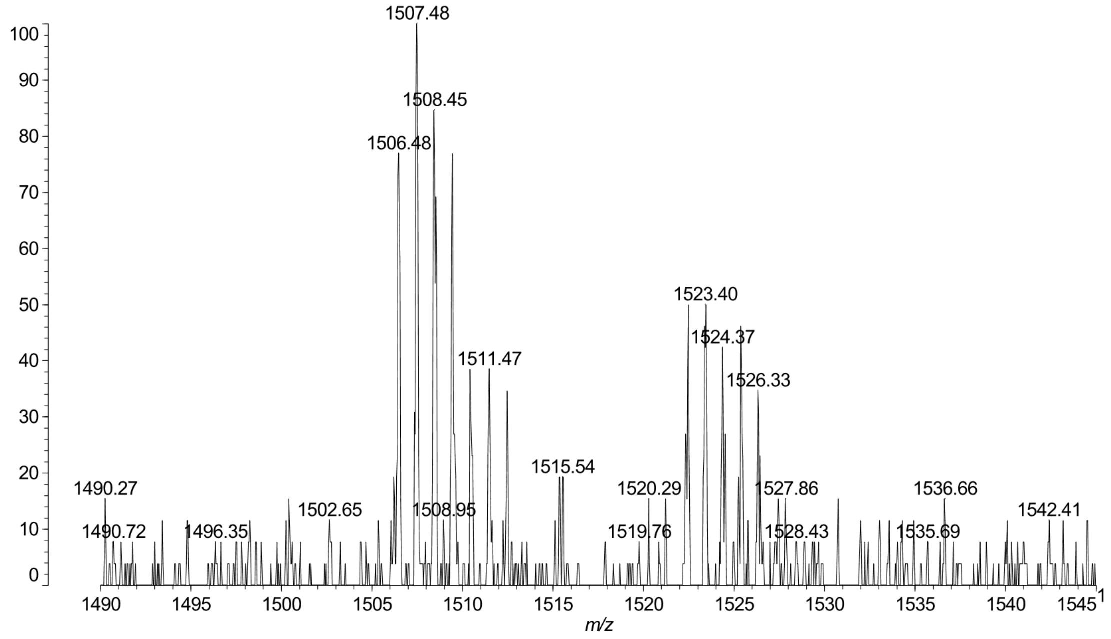

Figure S14: MALDI-TOF analysis for compound **5**

5. Thermogravimetric analyses for compounds 4 and 5

Table S1. Thermal properties for compounds 4 and 5 under N_2

Compounds	Si+O ratio (%)	T_{sub} (°C)	Td_5 (°C)	Residue at 1000 °C (%)
**4**	46.28	> 1000	153	18
**5**	33.43	> 1000	322	21

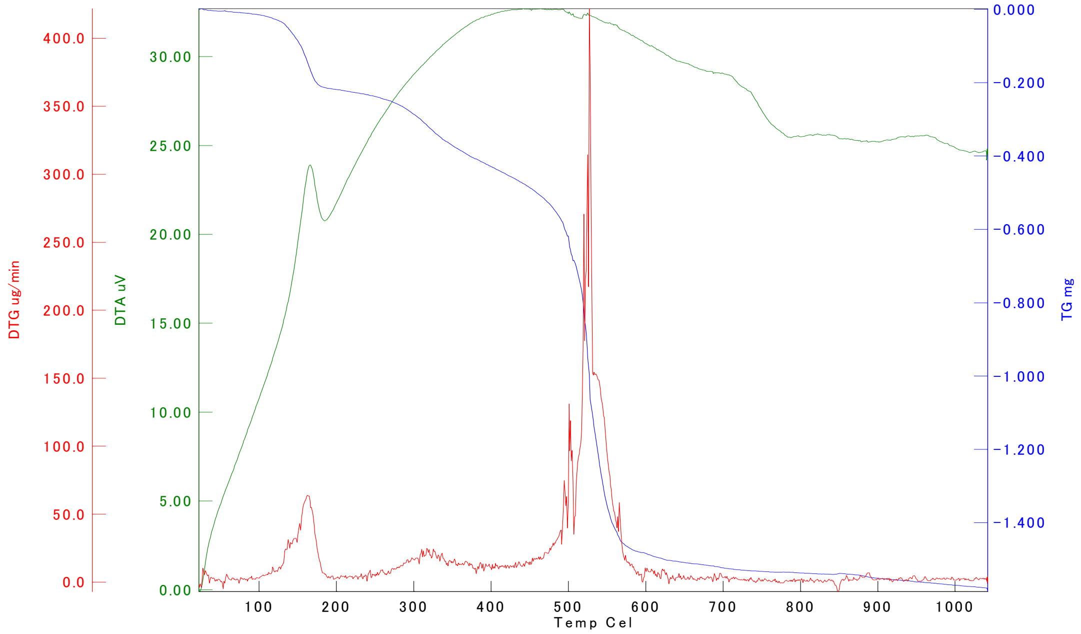

Figure S15: Thermogravimetric graphic of compound **4**

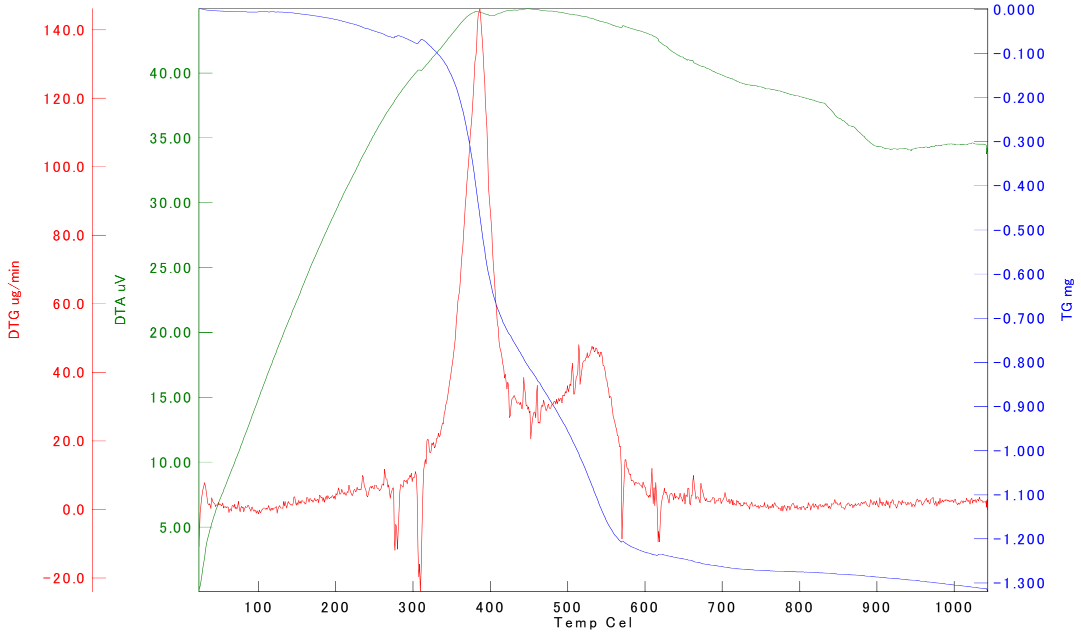

Figure S16: Thermogravimetric graphic of compound **5**
